# Phase I and pharmacological study of the broad-spectrum tyrosine kinase inhibitor JNJ-26483327 in patients with advanced solid tumours

**DOI:** 10.1038/sj.bjc.6605867

**Published:** 2010-09-07

**Authors:** I R H M Konings, M J A de Jonge, H Burger, A van der Gaast, L E C van Beijsterveldt, H Winkler, J Verweij, Z Yuan, P Hellemans, F A L M Eskens

**Affiliations:** 1 Department of Medical Oncology, Erasmus University Medical Center, Room HE-118, P.O. Box 2040, Rotterdam 3000 CA, The Netherlands; 2 Ortho Biotech Oncology Research and Development, Turnhoutseweg 30, Beerse 2340, Belgium

**Keywords:** phase I, JNJ-26483327, pharmacokinetics, pharmacodynamics, tyrosine kinase inhibitor

## Abstract

**Background:**

JNJ-26483327 is an oral, potent, multi-targeted tyrosine kinase inhibitor, inhibiting kinases of epidermal growth factor receptor (EGFR)-1, -2 and -4, rearranged during transfection (RET) receptor, vascular endothelial growth factor receptor (VEGFR)-3 and Src family (Lyn, Fyn, Yes) at low nanomolar concentrations. This phase I, accelerated titration study assessed maximum tolerated dose, safety, pharmacokinetics and pharmacodynamic effects of JNJ-26483327.

**Methods:**

Nineteen patients with advanced cancers received JNJ-26483327 continuous twice daily (BID) in escalating dose cohorts ranging from 100 to 2100 mg. Pharmacodynamic effects were assessed in paired skin biopsies and blood.

**Results:**

JNJ-26483327 was well tolerated in doses up to 1500 mg BID, with target-inhibition-related toxicity such as diarrhoea and skin rash, and other common reported toxicities being nausea, vomiting, anorexia and fatigue. At 2100 mg, two episodes of dose-limiting toxicity were observed, consisting of grade 3 anorexia and a combination of grade 3 anorexia and fatigue, respectively. Pharmacokinetics were dose proportional up to 1500 mg in which plasma levels were obtained showing anti-tumour activity in xenograft mouse models. Pharmacodynamic analysis did not show a substantial effect on expression of Ki-67, p27^kip1^, phosphorylated mitogen-activated protein kinase, phosphorylated Akt and EGFR, and serum levels of sVEGFR-2, VEGF-C and VEGF-D remained unchanged. Stable disease was noted in six patients (32%).

**Conclusion:**

JNJ-26483327 is well tolerated and shows a predictable pharmacokinetic profile; the recommended dose for further studies is 1500 mg BID.

Protein kinases represent over 2% of all human genes and ∼9% of known cancer genes. Overexpression or mutational and constitutive activity of these kinases plays an important role in the pathophysiology of tumours. Tyrosine kinase inhibitors (TKIs) have become an emerging new class of anti-cancer agents because of the importance of their targets in tumour proliferation, survival (apoptosis), tumour neoangiogenesis, invasion and metastasis (Blume-Jensen and Hunter, 2001; Shawyer *et al*, 2002). Significant clinical successes have meanwhile been achieved with relatively selective TKIs such as imatinib mesylate (Gleevec Glivec), erlotinib (Tarceva), gefitinib (Iressa) and lapatinib (Tyverb) (Demetri *et al*, 2002; Fukuoka *et al*, 2003; Geyer *et al*, 2006; Perez-Soler, 2007) as well as with the broad-spectrum TKIs sunitinib (Sutent) and sorafenib (Nexavar) (Llovet *et al*, 2008; Escudier *et al*, 2009; Motzer *et al*, 2009).

According to current understanding, most TKIs exert their activity in a cytostatic manner, suggesting that prolonged and maybe continuous treatment is to be recommended. Thus, an oral route of administration is of clear advantage, and an adequate oral bioavailability is a prerequisite (Eskens and Verweij, 2000). Toxicities observed with the use of TKIs appear to be linked to their primary mode of action (e.g. skin rash for EFGR inhibitors because of epidermal growth factor receptor (EGFR)-kinase inhibition in the basal layer of the skin), but other toxicities can occur for which no clear-cut pathophysiologic explanation exists as yet (Stadler, 2006).

JNJ-26483327 has displayed binding affinity to multiple tyrosine kinase receptors known to play a role in a wide variety of neoplasms. It showed potent (IC50 <10 nM) *in vitro* inhibitory activity against EGFR kinase, several mutationally activated EGFR kinases and against RET-receptor kinase. Inhibitory activity against vascular endothelial growth factor receptor (VEGFR)-3, Her4 and Src family (Lyn, Fyn, Yes) tyrosine kinases was shown over an IC50 range of 11–99 nM. Other tyrosine kinases inhibited by JNJ-26483327 (IC50 100–1000 nM) include c-Src, Her2, Flt3 and others. In preclinical studies, anti-cancer activity was observed and a favourable safety and tissue distribution profile was seen, including passage of the blood–brain barrier. JNJ-26483327 is administered as a twice daily (BID) oral regimen and has an oral bioavailability of ∼80% (Johnson & Johnson, data on file).

This first-in-man study was designed to determine safety, maximum tolerated dose (MTD) and dose-limiting toxicity (DLT) of JNJ-26483327 in patients with refractory or advanced solid tumours. In addition, the pharmacokinetic profile, pharmacodynamic activity in various surrogate tissues and preliminary signals of anti-tumour activity were assessed.

## Materials and methods

### Eligibility criteria

Patients with a histologically or cytologically confirmed diagnosis of advanced solid malignancy for whom no standard options existed or who were no longer responding to established treatments were eligible. Additional eligibility criteria included age ⩾18 years; ECOG performance ⩽2; life expectancy >3 months; adequate bone marrow function, without the support of cytokines and/or erythropoietin (white blood cell (WBC) count >3.0 × 10^9^ per l, absolute neutrophil count >1.5 × 10^9^ per l, platelet count >100 × 10^9^ per l, haemoglobin >10.0 g dl^–1^), hepatic function (total bilirubin level ⩽1.5 times institutional upper limit of normal (iULN), serum alanine transferase and aspartate aminotransferase ⩽2.5 times iULN or ⩽5 times iULN in case of liver metastases) and renal function (serum creatinine <1.5 times iULN); no chemotherapy, radiotherapy or immunotherapy within 28 days; no history of uncontrolled heart disease or arterial hypertension (systolic blood pressure ⩾160 mm Hg and/or diastolic blood pressure ⩾100 mm Hg despite appropriate medication). Specific exclusion criteria included a history of pulmonary fibrosis, known central nervous system metastases, impairment of gastrointestinal absorption status and inability to swallow. Left ventricular ejection fraction (LVEF) based on MUGA scan was required to be >50%. The study was approved by local ethics committees and all patients gave written informed consent before any study-related procedure.

### Study design

JNJ-26483327 was supplied by Johnson & Johnson Pharmaceutical Research and Development, Beerse, Belgium as 10 or 50 mg l^–1^ oral solution for doses ⩽1200 mg, or capsules of 50, 100 or 300 mg for doses ⩾1500 mg. Medication was taken in combination with food BID with 12 h intervals. A cycle was defined as 28 days of treatment.

The starting dose was 100 mg BID, which was selected based on preclinical data in rodents (one-third of the toxic dose low and one-tenth of the dose causing severe toxicity in rat) and a preceding study in healthy male volunteers. In the latter study, JNJ-26483327 was safe and well tolerated with single doses up to and including 200 mg. On the basis of the observed half-life, a BID-dosing regimen was chosen for this study.

The study followed an accelerated-escalation design. Initial cohorts consisted of one patient with dose doublings between cohorts in the absence of grade ⩾2 toxicity according to the National Cancer Institute Common Toxicity Criteria version 3.0 during the first cycle (http://ctep.cancer.gov/protocolDevelopment/electronic_applications/docs/ctcaev3.pdf). Once grade ⩾2 considered drug-related toxicity occurred during the first cycle, two additional patients were enrolled at that dose level with subsequent cohorts in accordance with a conventional dose-escalation (3+3) model. DLT was defined as any grade 3 or 4 non-haematological toxicity, except for nausea, vomiting or diarrhoea responsive to treatment; grade 3 skin rash/acne responsive to treatment, alopecia and isolated grade 3 GGT elevations; need for loperamide for >7 days to treat or prevent grade ⩾2 diarrhoea; need for continuous administration of 5HT-3 antagonists for >7 days to treat or prevent grade ⩾2 nausea or vomiting; a relative decrease of LVEF on MUGA scan of >20% compared with baseline; any grade 4 haematological toxicity or treatment interruptions of >14 days for grade ⩾2 toxicity in cycle 1.

If during the conventional escalation stage no DLT occurred, the dose for the next cohort was incremented with 20–100%. If DLT was observed during cycle 1 in one of three patients, an additional three patients were enrolled at that dose level. If ⩾2 out of 6 patients experienced DLT, the MTD was exceeded and three more patients were enrolled at the next lower dose level, unless already six patients had been accrued at that dose. The MTD was defined as the highest dose at which less than one-third of the patients experienced DLT.

No intrapatient dose escalation was allowed. In the absence of DLT or disease progression, patients were allowed to continue on JNJ-26483327 at the dose level assigned.

### Pretreatment and follow-up studies

Prior to therapy, a complete medical history was taken and a physical examination was performed. A complete blood cell (CBC) count, WBC count and differential and serum biochemistry including lipid profile and coagulation tests were performed, as were urinalysis, a 12-lead electrocardiogram (ECG) and MUGA scanning. Weekly evaluations during the first cycle, every other week during the second cycle and monthly thereafter included history, physical examination, adverse event assessment, CBC, WBC+differential, serum chemistry, ECG and urinalysis. An ECG was performed predose and 1, 2, 4, 8 and 24 h postdose on days 1 and 28. Follow-up of LVEF by MUGA scanning was performed at the end of the first and second cycle. Tumour measurements were performed at baseline and during every other cycle. Response was assessed according to RECIST (Therasse *et al*, 2000).

### Pharmacokinetic sampling and data analysis

For pharmacokinetic analysis, blood samples (3 ml) were collected before dosing and 0.5, 1, 2, 3, 4, 6, 8 and 24 h after drug administration on days 1 and 28 and predose, 0.5, 1, 2, 4 and 6 h postdose on day 15 of the first cycle and day 28 of the second cycle. Furthermore, trough PK samples were taken before drug administration on days 8 and 22. Blood samples were collected and protected from light in EDTA-Vacutainer^®^ tubes and plasma was separated by centrifugation and stored at −20°C within 2 h of collection. Plasma concentrations of JNJ-26483327 were determined by validated, selective and sensitive liquid chromatography–mass spectrometry bioanalytical method. The PK parameters gathered were area under the plasma concentration–time curve from time zero and extrapolated to infinity (area under the curve, AUC_inf_) after the first dose and calculated over the 12-h dose interval tau at steady state (AUC_tau_), maximum observed plasma concentration (*C*_max_), time to reach *C*_max_ (*t*_max_), apparent terminal half-life (*t*_1/2_), apparent total plasma clearance (CL/F) and apparent volume of distribution (Vdz/F).

Graphical analysis was performed using dose-normalised *C*_max_ as well as AUC of days 1 and 28 for exploration of dose proportionality across doses.

### Pharmacodynamic assessments

Modulation of EGFR, phosphorylated mitogen-activated protein kinase (pMAPK), phosphorylated Akt (pAkt), Ki-67 (an indicator of cellular proliferation) and p27^KIP1^ (kinase inhibitory protein 1) was assessed in skin biopsies at screening and on day 28 of the first treatment cycle. Treatment-induced changes in these cellular biomarkers were examined using immunohistochemistry (IHC) on formalin-fixed and paraffin-embedded skin tissue sections. Punch biopsies (4 mm width × 4 mm depth) were taken from the lateral aspect of the upper extremity just before the first dose and at the end of cycle 1. Specimens were immediately fixed in 10% buffered neutral formalin for 16–24 h, and embedded in paraffin. Tissue sections (4 *μ*m) were mounted onto silan adhesive ‘Star Frost’ glass slides (Waldemar Knittel Glaser, Braunschweig, Germany), dried overnight at 50°C, deparaffinised and rehydrated.

Antibodies used were monoclonal mouse anti-human Ki-67 antigen (clone MIB-1, DakoCytomation, Glostrup, Denmark); monoclonal mouse anti-human p27^KIP1^-protein (clone 1B4, Monosan Sanbio, Uden, The Netherlands); phospho-p44/42 MAP kinase (Thr202/Tyr204) antibody (#9101, Cell Signaling Technology, Bioké, Leiden, The Netherlands); pAkt (Ser473) antibody (#3787, Cell Signaling Technology); monoclonal mouse anti-EGFR (clone E30, DakoCytomation). A pressure cooker-enhanced procedure was used for antigen retrieval, except EGFR, which required proteinase K treatment. Antigen detection and visualisation (Dako Envision+System-HRP, DakoCytomation, Glostrup, Denmark) were according to the standard staining procedures as provided by the manufacturer's instructions. Positive and negative control specimens were used for each IHC staining batch and, if available, an appropriate blocking peptide was included as well. Treatment effects of JNJ-26483327 on Ki-67 and p27^KIP1^ were assessed by counting ⩾1000 positive keratinocytes and expressing the markers as percentage. Regarding pMAPK-, pAkt- and EGFR-positive keratinocytes, the proportion of positive cells as well as the intensity of staining were estimated using the Allred scoring system (Allred *et al*, 1998).

Blood samples were taken on day 1 before the first dose and on days 15 and 28 in cycles 1 and 2. Blood was collected in a 6-ml serum separator tube (Vacutainer^®^ SST No. 367784, Becton Dickinson, Franklin Lakes, NJ, USA) and allowed to clot for 30 min before centrifugation at 1000 **g**. Serum was thereupon removed, aliquotted into three separate 3.6 ml cryotubes (Nunc, Cat. No. 366524, Thermo Fisher Scientific, Rochester, NY, USA) and stored at ⩽−20°C until further analysis. Treatment-induced changes in serum levels of sVEGFR-2, VEGF-C and VEGF-D were examined using specific Quantikine human VEGF Immunoassays (R&D systems Europe, Abingdon, UK; human sVEGFR-2 (DVR200), human VEGF-C (DVEC00), human VEGF-D (DVED00)). Absolute concentrations of serum VEGF proteins were determined according to the manufacturer's protocol, normalised to baseline levels and relative changes were correlated to the administered dose of JNJ-26483327.

### Statistical methods

The pharmacokinetic analysis and descriptive statistics were performed using PKAA 2.00 (developed for J&JPRD). PKAA 2.00 uses WinNonLin 5.2.1. Elimination rate constants (*λ*_z_) were calculated as the negative of the slope of the terminal log-linear segment of the plasma concentration–time curves. The range of data used for each subject and dose were determined by visual inspection of a semi-logarithmic plot of concentration *vs* time. At least three data points were used to estimate *λ*_z_. All PK calculations and figures used validated software.

## Results

Nineteen patients (16 male, 3 female), with a median age of 61 years (47–74), were enrolled between October 2006 and July 2008. Patient demographics, baseline characteristics and prior anti-cancer treatments are listed in Table 1.

A total of 44 cycles of JNJ-26483327 was administered, and the maximum number of cycles received was six (received by two subjects) with duration of treatment ranging from 11 to 168 days. Dose levels studied were 100 mg (*n*=1), 200 mg (*n*=1), 400 mg (*n*=1), 800 mg (*n*=3), 1200 mg (*n*=3), 1500 mg (*n*=6) and 2100 mg (*n*=4) (Table 2).

### Safety

Occurrence of side effects, as a function of schedule and dose, is listed in Table 3. Nausea, diarrhoea, vomiting, anorexia, fatigue and skin rash were principal toxicities of JNJ-26483327.

Diarrhoea occurred in >60% of patients at all but the lowest dose level was mostly mild or manageable with loperamide. One patient (dose level 1500 mg) experienced grade 3 diarrhoea that quickly resolved after loperamide initiation. Other common gastrointestinal side effects were nausea and, to a lesser extent, vomiting, which was usually mild and only required specific treatment in one patient. Eight patients complained of loss of appetite, with frequency increasing with dose. In two patients (dose level 2100 mg) grade 3 anorexia was observed.

**Figure 1:**
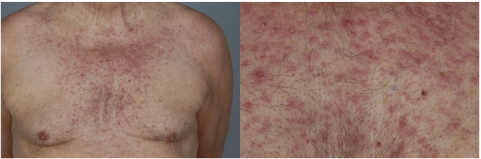
Skin toxicity after administration of JNJ-26483327.

Cutaneous side effects manifested mainly as rash, occurring in 11 out of 19 patients, with a hint of dose dependency (6 out of 11 received ⩾1500 mg) and predominantly located on the chest (Figure 1). Skin toxicity predominantly occurred during the first cycle and was transient with ongoing treatment. Skin discoloration, exfoliation and dry skin were other drug-induced skin toxicities.

Another frequently observed toxicity was mild fatigue (47.4% of patients). Haematological toxicity consisted of anaemia, with grade 2 anaemia detected in three patients. None of the subjects with paired LVEF measurements (*n*=15) showed a significant decrease in LVEF and no hypertension was noted.

### Dose-limiting toxicity

At 2100 mg, two episodes of DLT, consisting of grade 3 anorexia and of a combination of grade 3 anorexia and fatigue, were observed in one patient each. Other coinciding toxicities observed in the latter patient were grade 2 nausea, vomiting and diarrhoea, ultimately resulting in treatment interruption at day 11. All (four) patients in the 2100-mg cohort had substantial difficulty with the capsule load (seven capsules of 300 mg BID). On the basis of these combined observations, the dose of 2100 mg was considered to be intolerable and, therefore, the MTD was set at 1500 mg BID. At this dose, three additional patients were studied, none of whom experienced DLT.

### Pharmacokinetics

Plasma concentrations of JNJ-26483327 rapidly increased after oral administration. Maximum plasma concentrations (*C*_max_) were reached 2–3 h after the first administration of the liquid formulation (dose levels 800 and 1200 mg) and up to 4 h after administration of the capsule formulation (dose levels 1500 and 2100 mg). On day 1, *C*_max_ and overall drug exposure (AUC_last_) increased with increasing doses up to 1200 mg. At this dose level, peak concentrations were on average nearly 4 *μ*g ml^–1^. The 1500 and 2100 mg dose groups showed lower *C*_max_-values in the order of 2 *μ*g ml^–1^ as well as a decrease in overall drug exposure. After the peak, plasma concentrations declined biphasically. For all dose levels, the terminal half-life was 5–8 h.

**Figure 2:**
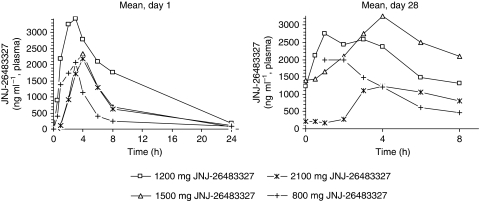
Mean plasma concentration–time profile of JNJ-26483327 after administration of JNJ-26483327 orally BID, days 1 and 28.

At steady state (as determined on days 15 and 28), *C*_max_ was reached 1–2 h after ingestion of liquid formulation and 2–5 h after administration of capsule formulation; *C*_max_ and AUC increased with increasing dose up to 1500 mg, but not beyond this dose. Dose-normalised *C*_max_ and AUC of 1200 mg liquid formulation and 1500 mg capsule formulation were comparable. Table 4 displays pharmacokinetic parameters of JNJ-26483327 for all dose levels on days 1 and 28 of cycle 1. Mean plasma concentration *vs* time curves of 1500 mg BID JNJ-26483327 on days 1 and 28 of cycle 1 are shown in Figure 2.

### Pharmacodynamics

Upon treatment with JNJ-26483327, no evident histopathological effects were observed in paired skin samples. Furthermore, no consistent changes in EGFR-associated cell-signalling biomarkers (EGFR, pMAPK or pAKT) and indicators of cellular differentiation (p27^KIP1^) and proliferation (Ki-67) were detected in keratinocytes of 16 paired skin biopsies. Baseline-normalised serum levels of sVEGFR-2, VEGF-C and VEGF-D were not affected by treatment.

### Anti-tumour activity

Six patients had stable disease lasting more than two cycles; the median number of cycles in these patients was four (range 3–6). One patient with prostate cancer treated at 800 mg BID and another patient with renal cell carcinoma treated at 1500 mg BID showed stable disease at the end of the fourth cycle. Stable disease did not continue beyond six cycles of treatment. There was no significant relationship between the occurrence of stable disease lasting more than two cycles and dose, even though three out of six patients received ⩾1500 mg JNJ-26483327 BID.

## Discussion

This study was the first-in-human study evaluating the feasibility of oral administration of JNJ-26483327 given continuously BID. Main objectives were to define its toxicity profile and MTD, as well as the pharmacokinetic and pharmacodynamic properties in man.

JNJ-26483327 was well tolerated up to 1500 mg BID, with most common reported toxicities being nausea, vomiting, diarrhoea, anorexia, fatigue and skin rash. Cutaneous and gastrointestinal side effects predominate in studies with EGFR TKIs (Loriot *et al*, 2008; Hartmann *et al*, 2009). The exact pathogenesis is still unclear, but as functional EGFR is crucial for maintaining integrity of the gastrointestinal mucosa, for normal development and physiology of the epidermis, as well as for mucosal repair, inhibiting EGFR activity likewise can induce mucocutaneous side effects (Lacouture, 2006; Playford *et al*, 2006; Li and Perez-Soler, 2009). The occurrence of these side effects can even be considered to be indicative for true target inhibition. In this study, gastrointestinal side effects showed a 95% incidence. Diarrhoea, nausea and vomiting occurred at most dose levels, but tended to be dose dependent. In those patients experiencing diarrhoea, loperamide treatment was always effective. Skin toxicity consisted of usually mild rash, dry skin, skin exfoliation, erythema, skin discoloration and paronychia with an overall incidence of 68%. The frequency and/or type of skin event was not clearly dose dependent and in all circumstances local treatment with moisturising ointments was sufficient in controlling these effects. No treatment interruption was indicated or felt necessary. Other observed side effects were also mild, never exceeding grade 2. As JNJ-26483327 is a Her2 inhibitor potentially inducing cardiotoxicity, special attention was given towards early recognition of this phenomenon (Perez, 2008). In this study, no signs of cardiac impairment or hypertension were observed.

Initially, JNJ-26483327 was administered as oral solution, but as high amounts of the vehiculum Captisol have been noted to induce soft stools or diarrhoea in man, a capsule formulation was introduced for the higher dose levels (⩾1200 mg). The maximum drug load of JNJ-26483327 was 300 mg per capsule, resulting in a substantial capsule intake at the higher dose levels. At 2100 mg BID grade 3 anorexia and a combination of grade 3 anorexia and fatigue yielded protocol-defined DLT in two patients, but because of substantial difficulty with capsule intake experienced by all other patients at this dose, capsule load at 2100 mg BID was considered dose limiting as well, albeit not defined by protocol. Therefore, the recommended phase 2 dose for JNJ-26483327 was set at 1500 mg BID.

Pharmacokinetic analysis showed rapid absorption with *C*_max_ reached on average 1–2 h earlier in case of the solution. With a half-life of 5–8 h, the drug is suitable for BID dosing. Up to 1500 mg, *C*_max_ and AUC were dose proportional at steady state (day 28) albeit with substantial inter- and intrapatient variability. This is a common phenomenon for many oral TKIs. Steady-state plasma concentrations at 1500 mg were in the active range as observed in mouse xenograft models. The PK data support the clinically guided conclusion that the recommended phase 2 dose should be 1500 mg BID, as the pharmacokinetic profile at 2100 mg BID showed lower overall drug exposure, probably because of a decrease in bioavailability. Very low concentrations were observed in one specific subject after 2100 mg administration, which could account for the overall effect observed at this dose level; of note is that this subject had taken carbamazepine, a known inducer of CYP3A4.

Somewhat to our surprise, no clear JNJ-26483327-induced pharmacodynamic effects were observed in surrogate tissues in this study. No consistent histopathological effects were observed in skin biopsies and various biomarkers of EGFR-signalling and serum levels of regulators of (tumour-)angiogenesis also remained essentially unchanged, even in patients experiencing obvious on-target side effects such as diarrhoea and skin rash. As skin toxicity is a predominant side effect of many specific EGFR-TKI (Li and Perez-Soler, 2009), the question here is whether any of the other target inhibiting effects acts as main driver of the therapeutic potential of JNJ-26483327. As JNJ-26483327 indeed targets multiple tyrosine kinases, this is a possible explanation. Another question is whether other parameters such as inflammatory cytokines (e.g. IL-1*β*, IL-6, IL-8), placental growth factor or circulating tumour cells should have been explored to gain better insight in pharmacodynamic alterations at the tumoural level (Jain *et al*, 2009). In addition, our observations again stress the importance of performing pharmacodynamic research in the most essential tissue available, being the tumour. Although taking repeated tumour biopsies will be inconvenient or even somewhat cumbersome for patients, restricting pharmacodynamic research to surrogate tissues might lead to disappointing or even incorrect conclusions.

In our study, 32% of the patients had stable disease for more than two cycles, with a maximum of six cycles over a dose range of 800–2100 mg BID. Although there was no statistical correlation between dose and frequency and/or duration of disease stabilisation, the observation was that at doses of and exceeding 1500 mg BID more prolonged disease stabilisation was observed. Some cases of prolonged stable disease were observed; however, because of the small numbers, no recommendation as to which tumour type could benefit most of JNJ-26483327 administration can be made. Most prolonged disease stabilisation was seen in two patients with prostate and renal cell cancer, respectively. If in future studies with JNJ-26483327 at these dose levels a better correlation between clinical outcome and pharmacodynamic assessment could be made, this would undoubtedly help in better understanding this broad-spectrum TKI.

In summary, JNJ-26483327 is a novel multi-targeted TKI. It is well tolerated at the recommended dose level of 1500 BID with only mild and reversible gastrointestinal and skin toxicity. At this dose, JNJ-26483327 shows a predictable pharmacokinetic profile. Further studies to establish its clinical antitumour activity are currently being considered.

## Figures and Tables

**Table 1:** Patient characteristics

Total	19
Male/female	16/3
Median age (years)	61
Range (years)	47–74
	
Prior systemic therapy	18
Prior radiotherapy	5
Prior surgery	12
	
*ECOG performance score*	
0	4
1	13
2	2
	
*Tumour type*	
Colorectal carcinoma	6
Oesophageal carcinoma	5
Prostate carcinoma	2
Hepatocellular carcinoma	1
Mesothelioma	1
Renal cell carcinoma	1
ACUP	1
Cholangiocarcinoma	1
Thyroid carcinoma	1

Abbreviation: ECOG=Eastern Cooperative Oncology Group.

**Table 2:** Dose escalation scheme, treatment duration

**Dose level (mg)**	**No. of patients**	**Total no. of cycles**	**No. of patients with DLT in cycle 1**
100	1	2	—
200	1	2	—
400	1	2	—
800	3	10	—
1200	3	4	—
1500	6	17	—
2100	4	7	2

Abbreviation: DLT=dose-limiting toxicity.

Administration scheme: JNJ 26483327 twice daily, continuously, 28 days per cycle.

**Table 3:** Principal JNJ-26483327-related side-effects

	**100 mg (*n*=1)**		**200 mg (*n*=1)**		**400 mg (*n*=1)**		**800 mg (*n*=3)**		**1200 mg (*n*=3)**		**1500 mg (*n*=6)**		**2100 mg (*n*=4)**		**Total (*n*=19)**	
	**G 1/2**	**G 3/4**	**G 1/2**	**G 3/4**	**G 1/2**	**G 3/4**	**G 1/2**	**G 3/4**	**G 1/2**	**G 3/4**	**G 1/2**	**G 3/4**	**G 1/2**	**G 3/4**	***n***	**%**
Nausea	0	0	0	0	1	0	2	0	3	0	6	0	1	0	13	68.4
Diarrhoea	0	0	1	0	1	0	2	0	2	0	3	1	2	0	12	63.2
Rash	1	0	1	0	0	0	2	0	1	0	3	1	2	0	11	57.9
Vomiting	0	0	0	0	1	0	2	0	2	0	3	0	2	0	10	52.6
Fatigue	1	0	0	0	0	0	1	0	1	0	3	0	2	1	9	47.4
Anorexia	0	0	0	0	0	0	1	0	1	0	3	0	1	2	8	42.1
Anaemia	0	0	1	0	1	0	0	0	1	0	0	0	0	0	3	15.8
Dysphonia	0	0	0	0	0	0	0	0	1	0	2	0	0	0	3	15.8
Pain	0	0	0	0	1	0	0	0	1	0	0	0	0	0	2	10.5
Dry skin	0	0	0	0	0	0	0	0	0	0	2	0	0	0	2	10.5
Constipation	0	0	0	0	0	0	0	0	1	0	1	0	0	0	2	10.5
Weight decreased	0	0	0	0	0	0	1	0	0	0	0	0	1	0	2	10.5
Dyspepsia	0	0	0	0	0	0	0	0	1	0	1	0	0	0	2	10.5
Dry skin	0	0	0	0	0	0	0	0	0	0	2	0	0	0	2	10.5
Skin exfoliation (palms)	0	0	0	0	0	0	0	0	0	0	1	0	0	0	1	5.3
Skin discolouration	0	0	1	0	0	0	0	0	0	0	0	0	0	0	1	5.3

**Table 4:** Pharmacokinetic parameters of JNJ-26483327 for days 1 and 28 (steady state)

**Doses (BID)**	**Day**	**Patients (*n*)**	**AUC (ng h ml^−1^)** ^**a**^	***C*_max_ (ng ml^−1^)**	***t*_max_ (*h*)**	***t*_1/2_ (*h*)**	**CL/F (l *h*^−1^)**	**Vdz/F (l)**
800 mg	1	3	10 621±5230	2210±593	2.03 (2.00–3.02)	8.4±1.8	86.3±33.5	1021±401
	28	2	10 623	2390	1.52 (1.00–2.03)		76.4	
1200 mg	1	3	35 554±22 086	3707±1927	3.00 (2.00–6.00)	5.2±1.7	47.4±35.3	342±212
	28	2	21 230	2750	1.00 (1.00–1.00)		88.4	
1500 mg	1	6	16 388±11 956	2361±1765	4.00 (2.00–4.03)	4.8±0.5	144±104	1038±799
	28	6	26 225±20 491	3375±2116	3.99 (3.00–12.00)		286±540	
2100 mg	1	4^a^	20 357±25 622	2213±2983	3.54 (2.00–4.08)	6.7±1.7	3055±5099	36 985±62 264
	28	2	8467	1236	5.00 (4.00–6.00)		3087	

*t*_max_, median (min–max); *C*_max_, AUC, *t*_1/2_, CL/F, Vdz/F: mean±s.d. s.d.=standard deviation, given when data of >2 subjects.

AUC_inf_ after the first dose and AUC_tau_ at steady state (day 28).

a *n*=3 for AUC_inf_, *t*_1/2_, CL/F and Vdz/F.

## References

[bib1] Allred DC, Harvey JM, Berardo M, Clark GM (1998) Prognostic and predictive factors in breast cancer by immunohistochemical analysis. Mod Pathol 11: 155–168 9504686

[bib2] Blume-Jensen P, Hunter T (2001) Oncogenic kinase signalling. Nature 411: 355–365 11357143 10.1038/35077225

[bib3] Demetri GD, von Mehren M, Blanke CD, Van den Abbeele AD, Eisenberg B, Roberts PJ, Heinrich MC, Tuveson DA, Singer S, Janicek M, Fletcher JA, Silverman SG, Silberman SL, Capdeville R, Kiese B, Peng B, Dimitrijevic S, Druker BJ, Corless C, Fletcher CD, Joensuu H. (2002) Efficacy and safety of imatinib mesylate in advanced gastrointestinal stromal tumours. N Engl J Med 347: 472–480 12181401 10.1056/NEJMoa020461

[bib4] Escudier B, Eisen T, Stadler WM, Szczylik C, Oudard S, Staehler M, Negrier S, Chevreau C, Desai AA, Rolland F, Demkow T, Hutson TE, Gore M, Anderson S, Hofilena G, Shan M, Pena C, Lathia C, Bukowski RM (2009) Sorafenib for treatment of renal cell carcinoma: final efficacy and safety results of the phase III treatment approaches in renal cancer global evaluation trial. J Clin Oncol 27: 3312–3318 19451442 10.1200/JCO.2008.19.5511

[bib5] Eskens F, Verweij J (2000) Clinical studies in the development of new anticancer agents exhibiting growth inhibition in models: facing the challenge of a proper study design. Crit Rev Oncol Hematol 34: 83–88 10799834 10.1016/s1040-8428(00)00055-x

[bib6] Fukuoka M, Yano S, Giaccone G, Tamura T, Nakagawa K, Douillard JY, Nishiwaki Y, Vansteenkiste J, Kudoh S, Rischin D, Eek R, Horai T, Noda K, Takata I, Smit E, Averbuch S, Macleod A, Feyereislova A, Dong RP, Baselga J (2003) Multi-institutional randomized phase II trial of gefinitib for previously treated patients with advanced non-small-cell lung cancer. J Clin Oncol 21: 2237–2246 12748244 10.1200/JCO.2003.10.038

[bib7] Geyer CE, Forster J, Lindquist D, Chan S, Romieu CG, Pienkowski T, Jagiello-Gruszfeld A, Crown J, Chan A, Kaufman B, Skarlos D, Campone M, Davidson N, Berger M, Oliva C, Rubin SD, Stein S, Cameron D (2006) Lapatinib plus capecitabine for HER2-positive advanced breast cancer. N Engl J Med 355: 2733–2743 17192538 10.1056/NEJMoa064320

[bib8] Hartmann JT, Haap M, Kopp HG, Lipp HP (2009) Tyrosine kinase inhibitors – a review on pharmacology, metabolism and side effects. Curr Drug Metab 10: 470–481 19689244 10.2174/138920009788897975

[bib9] Jain RK, Duda DG, Willet CG, Sahani DV, Zhu AX, Loeffler JS, Batchelor TT, Sorensen AG (2009) Biomarkers of response and resistance to antiangiogenic therapy. Nat Rev Clin Oncol 6: 327–338 19483739 10.1038/nrclinonc.2009.63 PMC3057433

[bib10] Lacouture ME (2006) Mechanisms of cutaneous toxicities to EFGR inhibitors. Nat Rev Cancer 6: 803–812 16990857 10.1038/nrc1970

[bib11] Li T, Perez-Soler R (2009) Skin toxicities associated with epidermal growth factor receptor inhibitors. Target Oncol 4: 107–119 19452131 10.1007/s11523-009-0114-0

[bib12] Llovet JM, Ricci S, Mazzaferro V, Hilgard P, Gane E, Blanc JF, de Oliveira AC, Santoro A, Raoul JL, Forner A, Schwartz M, Porta C, Zeuzem S, Bolondi L, Greten TF, Galle PR, Seitz JF, Borbath I, Häussinger D, Giannaris T, Shan M, Moscovici M, Voliotis D, Bruix J, SHARP Investigators Study Group (2008) Sorafenib in advanced hepatocellular carcinoma. N Engl J Med 24: 378–390 10.1056/NEJMoa0708857 18650514

[bib13] Loriot Y, Perlemuter G, Malka D, Penault-Llorca F, Boige V, Deutsch E, Massard C, Armand JP, Soria JC (2008) Drug insight: gastrointestinal and hepatic adverse effects of molecular-targeted agents in cancer therapy. Nat Clin Pract Oncol 5: 268–278 18349858 10.1038/ncponc1087

[bib14] Motzer RJ, Hutson TE, Tomczak P, Michaelson MD, Bukowski RM, Oudard S, Negrier S, Szczylik C, Pili R, Bjarnason GA, Garcia-del-Muro X, Sosman JA, Solska E, Wilding G, Thompson JA, Kim ST, Chen I, Huang X, Figlin RA (2009) Overall survival and updated results for sunitinib compared with interferon alfa in patients with metastatic renal cell carcinoma. J Clin Oncol 27: 3584–3590 19487381 10.1200/JCO.2008.20.1293 PMC3646307

[bib15] Perez EA (2008) Cardiac toxicity of ErbB2-targeted therapies: what do we know? Clin Breast Cancer 8(Suppl 3): S114–S120 18777950 10.3816/cbc.2008.s.007

[bib16] Perez-Soler R (2007) Erlotinib: recent clinical results and ongoing studies in non small cell lung cancer. Clin Cancer Res 13: s4589–s4592 17671146 10.1158/1078-0432.CCR-07-0541

[bib17] Playford R, Hanby A, Gschmeissner S, Peiffer L, Wright N, McGarrity T (2006) The epidermal growth factor receptor (EGF-R) is present on the basolateral, but not the apical, surface of enterocytes in the human gastrointestinal tract. Gut 39: 262–266 10.1136/gut.39.2.262 PMC1383309 8977341

[bib18] Shawyer LK, Slamon D, Ullrich A (2002) Smart drugs: tyrosine kinase inhibitors in cancer therapy. Cancer Cell 1: 117–123 12086869 10.1016/s1535-6108(02)00039-9

[bib19] Stadler WA (2006) New targets, therapies and toxicities: lessons to be learned. J Clin Oncol 24: 4–5 16314613 10.1200/JCO.2005.04.2408

[bib20] Therasse P, Arbuck SG, Eisenhauer EA, Wanders J, Kaplan RS, Rubinstein L, Verweij J, Van Glabbeke M, van Oosterom AT, Christian MC, Gwyther SG (2000) New guidelines to evaluate the response to treatment in solid tumours. European Organization for Research and Treatment of Cancer, National Cancer Institute of the United States, National Cancer Institute of Canada. J Natl Cancer Inst 92: 205–216 10655437 10.1093/jnci/92.3.205

